# Supplementary material for: Complexome profiling of the Chlamydomonas psb28 mutant reveals TEF5 as an early PSII assembly factor
Source: Plant Cell. 2025 Mar 17;37(6):koaf055. doi: 10.1093/plcell/koaf055 (PMC12164590; doi:10.1093/plcell/koaf055)
Supplement: koaf055_Supplementary_Data [file koaf055_supplementary_data.zip › psb28_interactive.zip!/plots/intensity/qualityPlots/peptides/CGL56_Cre05.g247450.t1.2.html]

### Cre05.g247450.t1.2

Trivial name: CGL56  
SeqCoverage [median]: 34.70  
Mass: 85.01 kDa  
  
 

| Sequence | MissedCleavage | Mass [Da] | wtIntensity | muIntensity | Score |
| --- | --- | --- | --- | --- | --- |
| AAALPADSVTFSPK | 0 | 1373.72 | 13899.65 | 48948.82 | 163.12 |
| AAAPAAIAGGAASAASAAAAPALAAPK | 0 | 2159.17 | 10271.94 | 35073.30 | 127.35 |
| AAAPAAIAGGAASAASAAAAPALAAPKPAAPK | 1 | 2623.44 | 1745.87 | 5871.06 | 54.17 |
| AAAPAPAASSAPAPAPAPAPAPAPAAAPSSASVQDSK | 0 | 3188.61 | 7957.07 | 7910.51 | 82.34 |
| AAAPAPAASSAPAPAPAPAPAPAPAAAPSSASVQDSKLQVK | 1 | 3656.92 | 1439.97 | 4145.02 | 54.93 |
| AEPAPKPAAAPAPAPAPK | 1 | 1650.91 | 10973.92 | 21726.61 | 100.43 |
| AESGILDLR | 0 | 972.52 | 5283.10 | 10629.94 | 102.51 |
| AESGILDLRK | 1 | 1100.62 | 2207.97 | 14276.77 | 90.15 |
| APVAASDTTASAAPAAAK | 0 | 1569.80 | 12788.91 | 15480.39 | 312.48 |
| APVAASDTTASAAPAAAKEK | 1 | 1826.94 | 2125.45 | 1318.15 | 156.13 |
| AYLDRYGGYSGDLAPAAVK | 1 | 1985.98 | 0.00 | 3491.68 | 73.11 |
| EDALVVDVR | 0 | 1014.53 | 8225.83 | 20966.37 | 155.07 |
| EKSPAAAPAPAK | 1 | 1136.62 | 1202.70 | 2492.77 | 94.77 |
| EKSPAAAPAPAKAEPAPKPAAAPAPAPAPK | 3 | 2769.52 | 2405.57 | 0.00 | 54.62 |
| ELLAREDALVVDVR | 1 | 1596.88 | 334.84 | 17047.02 | 66.00 |
| FSAPDLSSVK | 0 | 1049.54 | 17767.55 | 44779.30 | 124.92 |
| ISLPESAGSGSGSEGAGAGVTLPK | 0 | 2128.06 | 8062.73 | 25441.30 | 147.35 |
| PAAPKPAAPMIAPAPAK | 1 | 1597.90 | 1212.81 | 7417.07 | 127.76 |
| PAAPMIAPAPAK | 0 | 1133.63 | 875.97 | 3477.03 | 140.09 |
| SEAQRAESGILDLR | 1 | 1543.80 | 0.00 | 2166.47 | 48.53 |
| SEAQRAESGILDLRK | 2 | 1671.89 | 0.00 | 4737.98 | 95.54 |
| SPAAAPAPAK | 0 | 879.48 | 3361.27 | 6035.45 | 126.52 |
| SPAAAPAPAKAEPAPKPAAAPAPAPAPK | 2 | 2512.38 | 5702.60 | 17564.72 | 58.27 |
| VEIPSFK | 0 | 818.45 | 3858.52 | 20609.12 | 105.39 |
| VKPGSSAPSFEAPSFSMPDFSGVQLPK | 1 | 2793.37 | 0.00 | 8419.83 | 69.28 |
| VSTPLTK | 0 | 744.44 | 6228.91 | 10725.38 | 128.57 |
| VSVAPEGTASTSD | 0 | 1219.56 | 4324.95 | 4608.64 | 87.81 |
| VVIMNDKDDVAAR | 1 | 1444.73 | 16896.14 | 36093.98 | 144.57 |
| VVIMNDKDDVAARDLAR | 2 | 1899.98 | 0.00 | 1927.83 | 68.26 |
| YGGYSGDLAPAAVK | 0 | 1367.67 | 11409.16 | 20462.52 | 225.22 |
| YLEMTSK | 0 | 870.42 | 4854.62 | 14602.45 | 145.04 |
| YRAAALPADSVTFSPK | 1 | 1692.88 | 623.58 | 3109.69 | 75.02 |
|  |  |  | 166041.6 | 441557.1 |  |
